# The hydroxyl moiety on carbon one (C1) in the monoterpene nucleus of thymol is indispensable for anti-bacterial effect of thymol

**DOI:** 10.1016/j.heliyon.2020.e03492

**Published:** 2020-03-17

**Authors:** Alex Boye, Justice Kwaku Addo, Desmond Omane Acheampong, Ama Kyeraa Thomford, Emmanuel Asante, Regina Elorm Amoaning, Dominic Nkwantabisa Kuma

**Affiliations:** a Department of Medical Laboratory Science, School of Allied Health Sciences, College of Health and Allied Sciences, University of Cape Coast, Cape Coast, Ghana; b Department of Chemistry, School of Physical Sciences, University of Cape Coast, Cape Coast, Ghana; c Department of Biomedical Science, School of Allied Health Sciences, College of Health and Allied Sciences, University of Cape Coast, Cape Coast, Ghana

**Keywords:** Ester-substitution, Ether-substitution, *E. coli*, Essential oils, Monoterpenes, *P. aeruginosa*, *S. aureus*, Structure activity relation, Thymol, Food toxicology, Biological sciences, Ethnopharmacology, Toxicology, Health sciences, Pharmacology

## Abstract

**Background:**

Thymol, a natural monoterpene phenol is not only relevant clinically as an anti-microbial, anti-oxidant and anti-inflammatory agent but also holds the prospect as a natural template for pharmaceutical semi-synthesis of therapeutic agents. It is a major component of essential oils from many plants. Evidence abound linking overall bioactivity of thymol to its monoterpene nucleus, specifically, the hydroxyl (-OH) substituent on carbon number one (C1) on the monoterpene nucleus. Other studies have posited that the overall bioactivity of thymol is not substantially altered by chemical modification of - OH on the C1 of the monoterpene nucleus. In view of this, it is still unclear as to whether removal or modification of the –OH on C1 of the monoterpene nucleus relates generally or context-dependently to bioactivity of thymol.

**Objective:**

The present study investigated anti-bacterial effects of ester-and-ether substituted derivatives of thymol on *S. aureus*, *P. aeruginosa* and *E. coli*.

**Materials and methods:**

twelve ester-and-ether substituted derivatives of thymol (6TM1s and 6TM2s) were synthesized and characterized by using HPLC, Mass spectrometry, and IR techniques. Anti-bacterial activity of the 12 thymol derivatives was evaluated using broth macrodilution and turbidimetric methods against pure clinical isolates (*S. aureus*, *P. aeruginosa* and *E. coli*). Standard anti-biotics used were Thymol Streptomycin and flucloxacillin, while DMSO was used as vehicle for thymol derivatives. MIC and MBC were determined.

**Results:**

Thymol produced broad-spectrum growth inhibition on all isolates. At equimolar concentrations, thymol and reference drugs produced concentration-dependent growth inhibition against the isolates (*Staphylococcus aureus*, *Pseudomonas aeruginosa* and *Escherichia coli*) compared to DMSO. Although the growth inhibitory effects of the ester-and-ether derivatives of thymol was significant (P ≤ 0.05) compared to DMSO, it was however insignificant (P ≥ 0.05) compared to thymol and reference antibiotics. Comparatively, at equimolar concentrations, ester-substituted derivatives of thymol, particularly the branched chain derivative (TM1C) produced more effective growth inhibition on the isolates than the ether-substituted derivatives of thymol. Thymol was twice as potent (MIC and MBC, 500 μg/ml) than both ester-and-ether substituted derivatives of thymol (MIC and MBC, > 1000 μg/ml) on all the three clinical isolates. Increase in side chain bulkiness of –OH moiety on the monoterpene nucleus of thymol decreased growth inhibition on isolates.

**Conclusion:**

Thymol has demonstrated broad-spectrum anti-bacterial effects attributable to the hydroxyl moiety on C1 of the monoterpene nucleus. Structural modification of the hydroxyl moiety on C1 of the monoterpene nucleus of thymol with either ether-or-ester substitutions yielded no significant anti-bacterial effects.

## 1 Introduction

Plant-based natural products continue to serve as templates for pharmaceutical semi-synthesis [1, 2, 3]. Plants contain many useful medicinal components including essential oils (EOs). Essential oils are a composite of many odorous and volatile compounds [4] and are useful for mankind in many capacities including pharmaceuticals, agrochemicals, and as aromatherapy and flavoring agents [4, 5]. Essential oils derived from plants are not only diverse in terms of their composition, but also exert many pharmacological effects including anti-microbial, anti-oxidant, and anti-inflammatory activities [5, 6, 7] and these biological and pharmacological properties are related to the type and chemistry of the constituent compounds [4]. Phenolic compounds represent a major component of EOs, and they contain the terpene nucleus. The terpenoids may exist in EOs as hemiterpene, monoterpene, or sesquiterpene [8]. Terpenes are active against Gram positive and Gram negative bacteria as well as fungi [9]. For instance, anti-microbial activity of EOs was attributed to oxygenated terpenoids and their hydrocarbon chain [10]. In some specific organisms such as *S. aureus*, EOs were shown to be four times potent than conventional anti-biotics such as chloramphenicol [11]. Essential oils by their lipophilic nature traverse cell membranes to interact with cellular components. It was shown that EOs easily traverses cell membranes of pathogenic bacteria cells to disrupt membrane structure and function, cellular damage and cause cell death [12, 13].

One major component of EOs with demonstrated potential for therapeutic application is thymol (2-isopropyl-5-methylphenol). Thymol is readily sourced from EOs of many plants including *Thymus vulgaris* [14], *Lippia gracilis* and *Lippia sidoides* [15, 16]. It is a natural monoterpene phenol and has many biological activities [15] for which reason it enjoys therapeutic application. For example, thymol is used as a wound dressing agent [17], varnish [18], food preservative [19], as an oral base [20] and as anti-bacterial agent [21]. Monoterpenes have been shown to disrupt membrane integrity; therefore anti-microbial effects of monoterpene-rich EOs are normally attributed to their terpene components [8]. For instance, perturbation of lipid fraction of plasma membrane was proposed as the mechanism by which monoterpene phenols such as thymol exerts their bactericidal effects [8].

Evidence abound that generally attribute the overall biological activity of monoterpene phenols such as thymol to the hydroxyl (-OH) substituent on carbon number one (C1) on the monoterpene nucleus [22, 23, 24]. Others studies have also demonstrated that the overall bioactivity of monoterpene phenols is not altered by the presence or absence or changing the position of the hydroxyl moiety (-OH) on the monoterpene nucleus [24, 25, 26]. To ascertain whether modification of the hydroxyl moiety on thymol relate to its anti-bacterial activity, this study focused on structural modification of the hydroxyl moiety of thymol with either ether-or-ester functional groups having varying hydrocarbon chains (straight, branched or aromatic). Specifically, structure activity relations (SAR) of the ether-and-ester derivatives of thymol were assessed relative to thymol and reference antibiotics (streptomycin and flucoxacillin) by determining % inhibition of growth of three pure clinical isolates (*S. aureus*, *P. aeruginosa* and *E. coli*) as well as determination of minimum inhibitory concentration (MIC) and minimum bactericidal concentration (MBC). Interestingly, two out of twelve thymol derivatives, one each of ether and ester derivatives showed anti-bacterial activity though not as effective as thymol or the reference antibiotics.

## 2 Materials and methods

### 2.1 Chemicals

Chemicals and reagents used in the study included thymol (Sigma Aldrich, St. Louis, Missouri, United States), Isopropyl alcohol and Dimethyl sulphoxide (DMSO), Flucloxacillin (Aurobindo Pharma limited, India) and Streptomycin sulphate (Merck Pharma Ltd, Ferozeshah Road, Delhi, India). All other chemicals and reagents used in the study were of analytical grade and were obtained from Sigma Aldrich.

### 2.2 Synthesis of ether-substituted derivatives of thymol

**Scheme 1:**
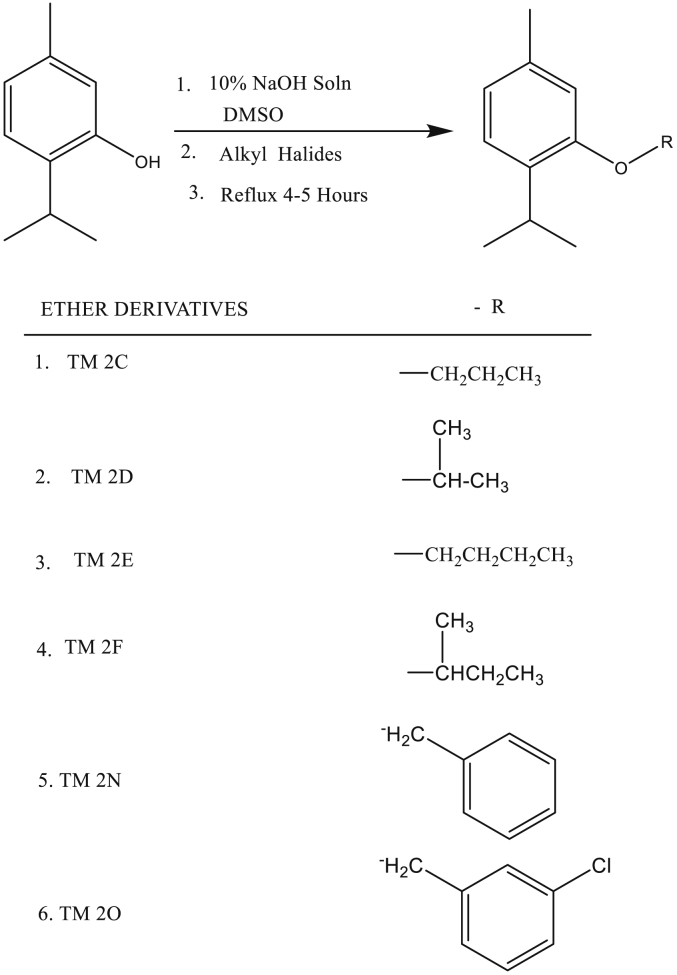
Synthesis of ether derivatives of thymol.

Ether-substituted derivatives of thymol were synthesized (Scheme 1) by using Williamson etherification reaction as previously described [27]. Briefly, sodium salt of thymol was prepared by dissolving thymol (1.00 g, 0.005 M) in 10% NaOH (10 ml) for 10 min with continuous stirring using a magnetic stirrer. DMSO (10 ml) was then added. The required stoichiometric amount of the various alkyl halides (0.005 M) was added slowly with stirring. The reaction mixture was then refluxed for about 4–5 h. Progress of the reaction was monitored by TLC and GC analysis. After completion, the reaction mixture was cooled to room temperature and the oily product was extracted with Dichloromethane (DCM), three times (3 × 10 ml). The combined DCM extract was transferred into a separatory funnel and washed with saturated sodium bicarbonate solution, two times (2 × 10 ml) and distilled water, two times (2 × 10 ml). The recovered DCM layer was dried over anhydrous sodium sulphate. This was then filtered to obtain the DCM solution containing the ether derivatives. The DCM was removed by concentrating on a rotary evaporator to obtain the crude ether derivatives. The crude products were purified by C.C using silica gel (60–120 mesh) with Hexane/Ethyl acetate (21:1 v/v) as eluent in an increasing polarity of the solvent system to afford pure ether derivatives (TM 2C, 2D, 2E, 2F, 2N, and 2O) in 80–90% yields.

### 2.3 Synthesis of ester-substituted derivatives of thymol

Ester-substituted derivatives of thymol were synthesized by adopting the method previously described [28] with some modifications. Briefly, a solution of sodium salt of thymol (1.00 g) was prepared by dissolving it in 10 ml of 10 % NaOH solution with continuous stirring using a magnetic stirrer for about ten minutes. To a solution of the prepared thymol (1.00equiv.) and trimethylamine (1.1equiv.) in anhydrous DCM, stoichiometric amount of Acid Chlorides were added at 0 °C for about 1 h. Stirring of the reaction mixture was continued at room temperature for about 8–10 h. Progress of reaction was monitored by thin layer chromatography (TLC) and gas chromatography (GC). After completion, the reaction mixture was quenched with distilled water and extracted with DCM (10 ml) three times. Finally, the combined organic layer (DCM layer) was washed with distilled water, brine and dried over anhydrous sodium sulphate, Na_2_SO_4._ After removal of the solvent in vacuo, the residue was purified by C.C using silica gel 60–120 mesh with Hexane/Diethyl ether (19:1 v/v) as eluent to afford pure ester derivatives (TM 1C, 1D, 1E, 1F, 1I, and 1Q) in 90–95 % yields. Both ether and ester derivatives of thymol were stored in a refrigerator at -18 ± 2 °C until use.

### 2.4 Mass spectrometry (MS) analysis

The MS spectra were acquired in both the negative and positive modes and full scan mass spectra were acquired from the mass-to-charge ratio (m/z) of 100–800. Parameters for the MS were as follows: Aux sweep gas 5au, ion spray voltage 5kV; capillary temperature 280^O^C.

### 2.5 Infra-red (IR) analysis of ether-and-ester substituted derivatives of thymol

FT-IR spectrophotometer (Perkin Elmer spectrum BX-II apparatus) was used to identify the characteristic functional groups in the thymol derivatives. Briefly, the IR spectra were obtained at ambient temperature. The background correction was established by determining IR spectrum of de-ionized water as a reference in similar conditions. Samples were scanned from 400-4000 cm^−1^ for 6 times in order to enhance signal to noise ratio.

### 2.6 Acquisition, storage and use of isolates

Three clinically relevant pure culture isolates were obtained from the Cape Coast Teaching Hospital (CCTH), Cape Coast, Ghana and were transported to the laboratory of the Department of Medical Sciences under the required conditions and biosafety measures. The isolates included: *Staphylococcus aureus* (Gram Positive), *Escherichia coli* (Gram negative Lactose Fermenter) and *Pseudomonas aeruginosa* (Gram negative Non-Lactose Fermenter). Isolates were stored in sterile Peptone broths at a temperature of 4 ± 2 °C. Isolates identity were confirmed prior to the experiments by using analytical profile index (bioMerieux API®) biochemical test strips for gram positive and gram negative bacteria. The Clinical and Laboratory Standards Institute (CLSI) guideline document (M07-A9 Vol. 32 No.2) that addresses reference methods for the determination of minimal inhibitory concentration (MIC) of aerobic bacteria by broth macrodilution, broth microdilution, and agar dilution was adopted for the study.

### 2.7 Preparation of drug dilutions

A stock solution (20,000 μg/ml) was prepared for each of the twelve (12) thymol derivatives (TM2N, TM1I, TM1D, TM1F, TM1C, TM1E, TM2C, TM2D, TM2E, TM2F, TM2O, and TM1Q), Thymol, DMSO, FLX and STR. With the exception of reference drugs all other drugs were solubilized in DMSO and diluted to the final concentration with sterile distilled water. DMSO, FLX and STR were however, both solubilized and diluted with sterile distilled water. Stock solutions were kept frozen at -18 ± 2 °C and thawed once when ready for use. Final drug concentrations of 62.5, 125, 250, 500 and 1000 μg/ml were respectively prepared in Muller Hinton broth (MHB) from the stock solution of each drug. These dilutions were prepared a step higher (double) than the final concentrations to compensate for the addition of an equal volume of the inoculum. Thus, 125 μg/ml (62.5 μg/ml), 250 μg/ml (125 μg/ml), 500 μg/ml (250 μg/ml), 1000 μg/ml (500 μg/ml) and 2000 μg/ml (1000 μg/ml).

### 2.8 Preparation of inoculum

Fresh colonies of pure cultures were obtained by subculture on Blood agar (BA) plate after 18–24 h aerobic incubation at 35 ± 2 °C. Inocula equivalent to 0.5 McFarland standards (1–2 × 10^8^ colony-forming units (CFU)/ml) were prepared for the three isolates. A final inoculum of 5 × 10^5^ CFU/ml required for the BMD was achieved first by 1:150 dilutions in MHB medium for each isolate. The subsequent 1:2 dilution with the final drug dilutions as described above brought the final required inoculum to 5 × 10^5^ CFU/ml as previously described [29].

### 2.9 Broth macrodilution procedure

Broth dilutions were done according to a previous method [29] with some modifications. Briefly, for each isolate, sufficient number of 75 × 25 mm sterile test tubes was arranged in rows and labeled for each drug to cover the range of concentrations in triplicate. Tubes were labeled with the respective concentrations (62.5, 125, 250, 500 and 1000 μg/ml) of each drug and the name of the isolate. A volume of 1 ml of each drug dilution in broth (the double concentrations) was added to the respective tubes. Aliquots of 1ml of the inoculum suspension of the particular isolate were then added to the contents of the tubes and mixed thoroughly. Turbidity and sterile control tubes of drug-free broth were included in each set-up. The turbidity control tube (contained 1 ml of drug-free broth and 1 ml of inoculum suspension) was used to control the adequacy of the broth to support the growth of the organism. The sterile control tube (2 ml of drug-free broth) was used to check the sterility of the broth prepared. All tubes were incubated at 35 ± 2 °C under aerobic condition overnight.

### 2.10 Determination of % inhibition

The turbidity (optical density, OD) controls for each isolate were measured spectrophotometrically at 450 nm by using Shimadzu spectrophotometer (UVmini-1240). Subsequently, OD of each isolate incubated with each drug was measured at 450 nm. Sterility control was used as the blank and the turbidity controls as the positive control. Experiments were repeated three times. % inhibition of growth was estimated by using the formula below:

% inhibition = [1 – A_t_ / A_C_] × 100

Where A_t_ = Absorbance of test sample

A_c_ = Absorbance of control

In all readings, a blank for both control and sample (test) were used but were auto-zeroed in the Spectrophotometer or by subtraction before recording actual A_t_ or A_c_.

### 2.11 Determination of minimum inhibitory concentration (MIC) and minimum bactericidal concentration (MBC)

MIC and MBC were determined as previously described [30, 31]. Briefly, by visible inspection by three independent microbiologists, each row of tubes for each drug and isolate for a particular batch were checked for inhibition of growth by comparing to the sterile control tube of that set-up. The lowest concentration tubes without visible growth after the overnight incubation were recorded as the MIC for that drug against the specific isolate. MIC tubes and other tubes that showed no visible growth were plated on blood agar (BA) plates and incubated at 35 ± 2 °C under aerobic condition overnight to check for MBC as previously described [31]. Tubes that showed growth were serially diluted 1:1000 and 100 μl of the dilution was plated on Plate Count agar (PCA) plates, incubated at 35 ± 2 °C under aerobic condition for 18–24 h. The number of colony forming units per milliliter (CFU/ml) was determined and these were compared to that of the turbidity control tubes of the respective isolates.

### 2.12 Statistical analysis

Data are expressed as mean ± SD, n = 3 experiments. Graph Pad Prism version 6 (Graph Pad Software, San Diego, CA, USA) was used in all statistical analyses. OD of each isolate growth before and after incubation with increasing concentrations of respective drugs was used to calculate % inhibition of growth. One Way ANOVA was used for multiple comparison of group means, followed by Tukey's post hoc test. P ≤ 0.05 was considered statistically significant in all analyses.

## 3 Results

### 3.1 HPLC results on thymol derivatives

**Figure 1:**
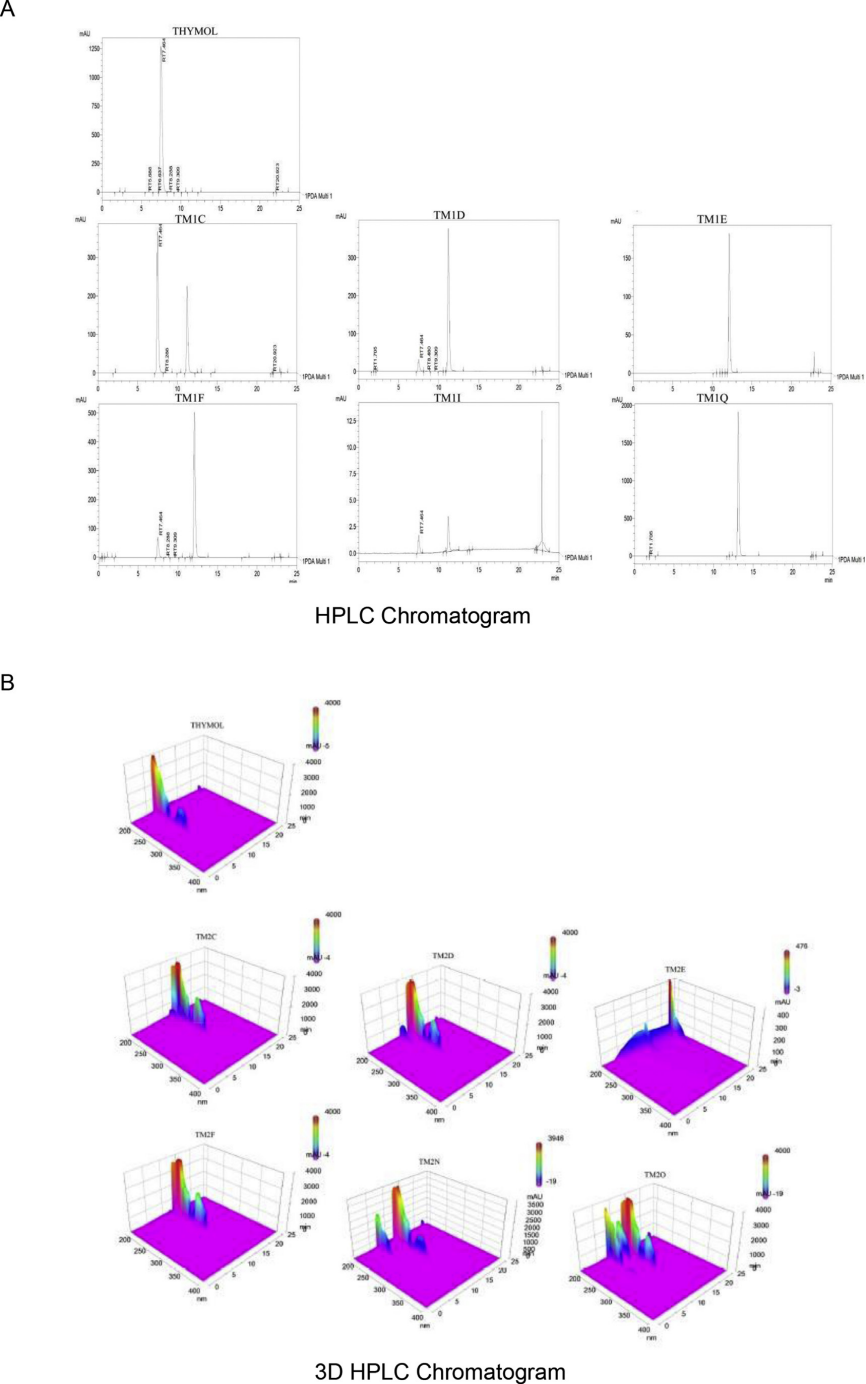
HPLC chromatogram of thymol and ether-substituted derivatives of thymol. (A) HPLC chromatogram of thymol and ether-substituted derivatives of thymol, and (B) A 3-D HPLC chromatogram of thymol and ether-substituted derivatives of thymol. HPLC - High performance liquid chromatography.

**Figure 2:**
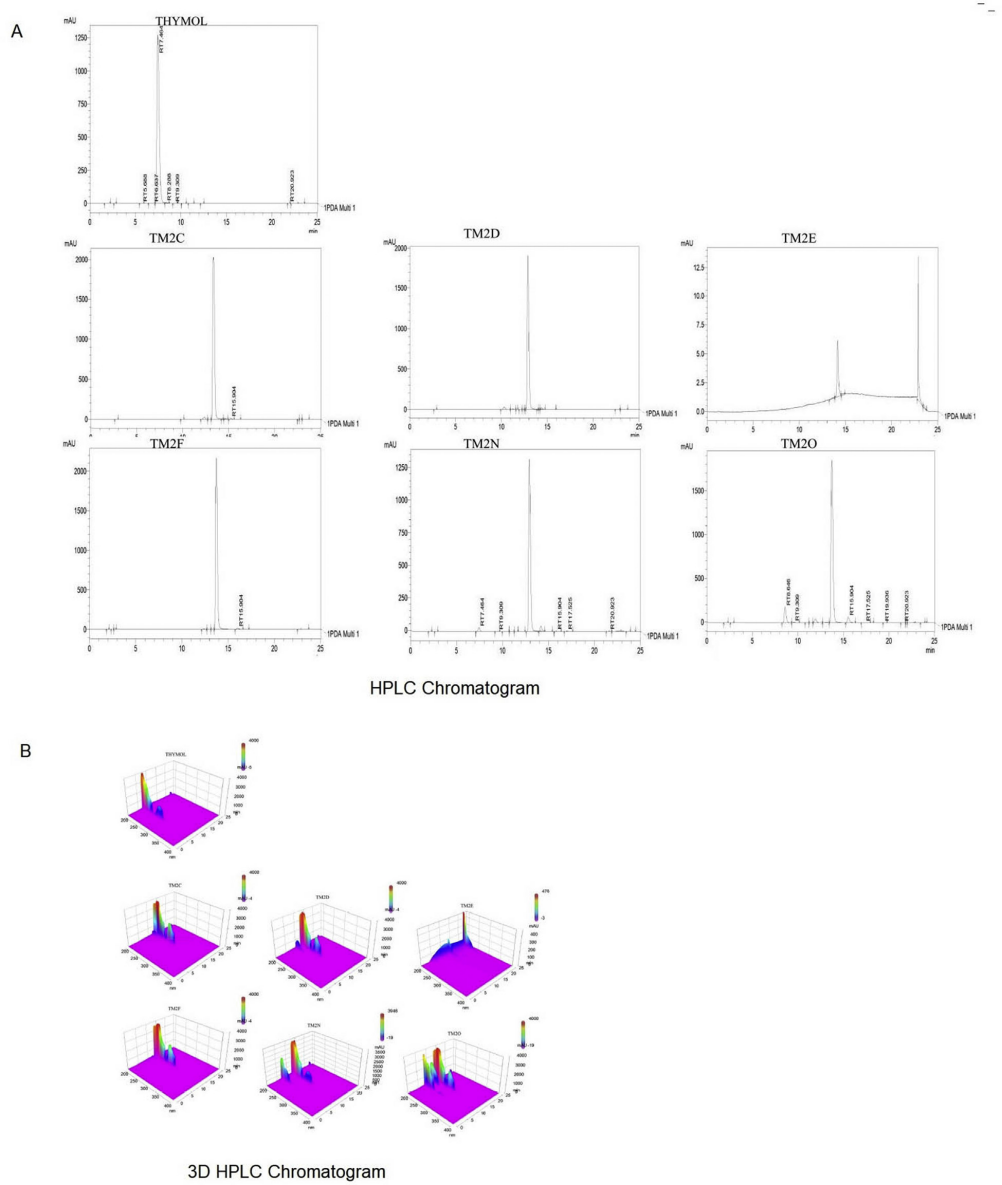
HPLC chromatogram of thymol and ester-substituted derivatives of thymol. (A) HPLC chromatogram of thymol and ester-substituted derivatives of thymol, and (B) A 3-D HPLC chromatogram of thymol and ester-substituted derivatives of thymol. HPLC - High performance liquid chromatography.

The relative purity of the ether-and-ester substituted derivatives of thymol is shown in Figures 1 and 2.

### 3.2 Structural elucidation of ether-and-ester substituted derivatives of thymol by IR analysis

**Figure 3:**
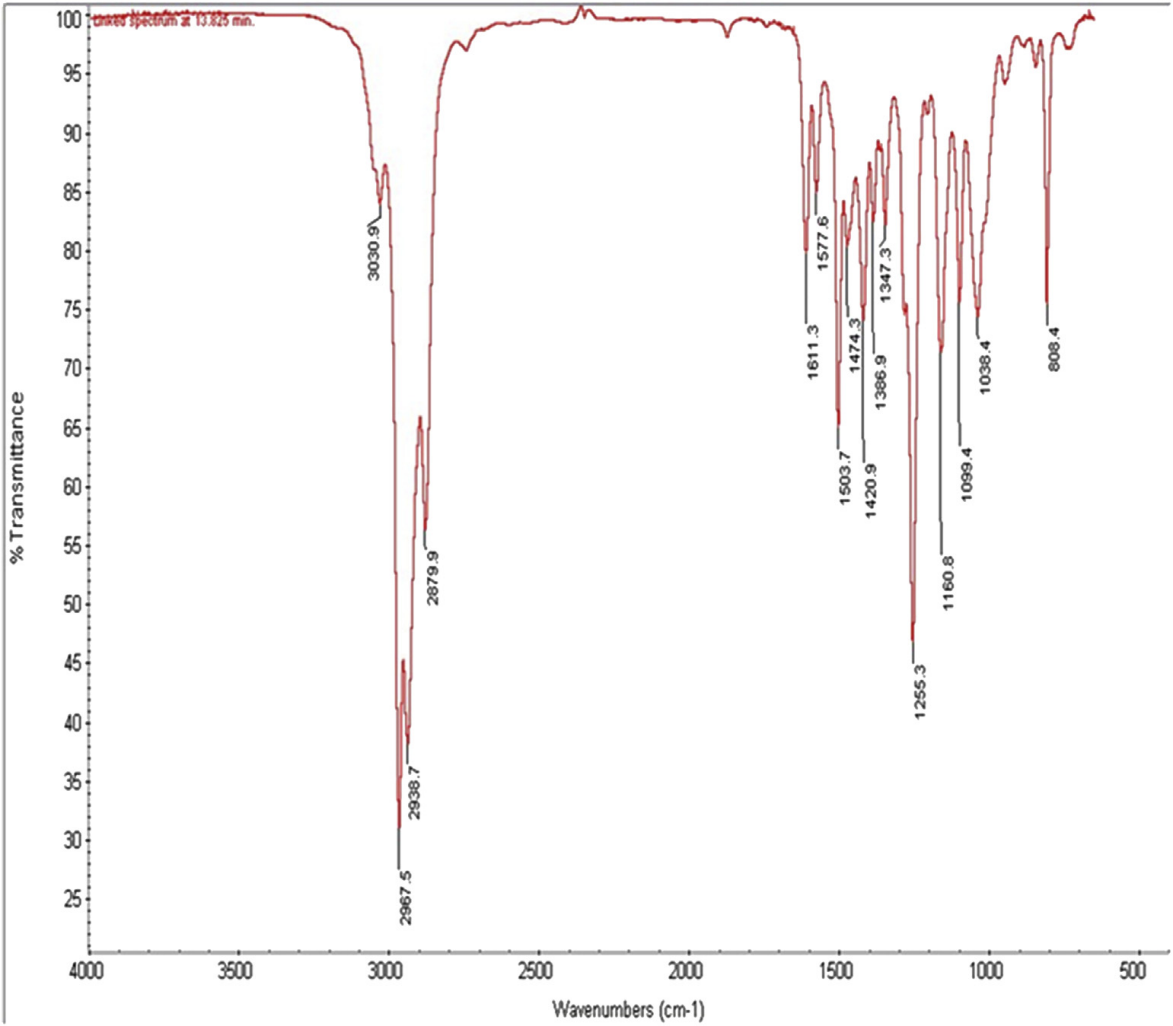
Infra-red (IR) spectra of ether-substituted derivatives of thymol.

**Figure 4:**
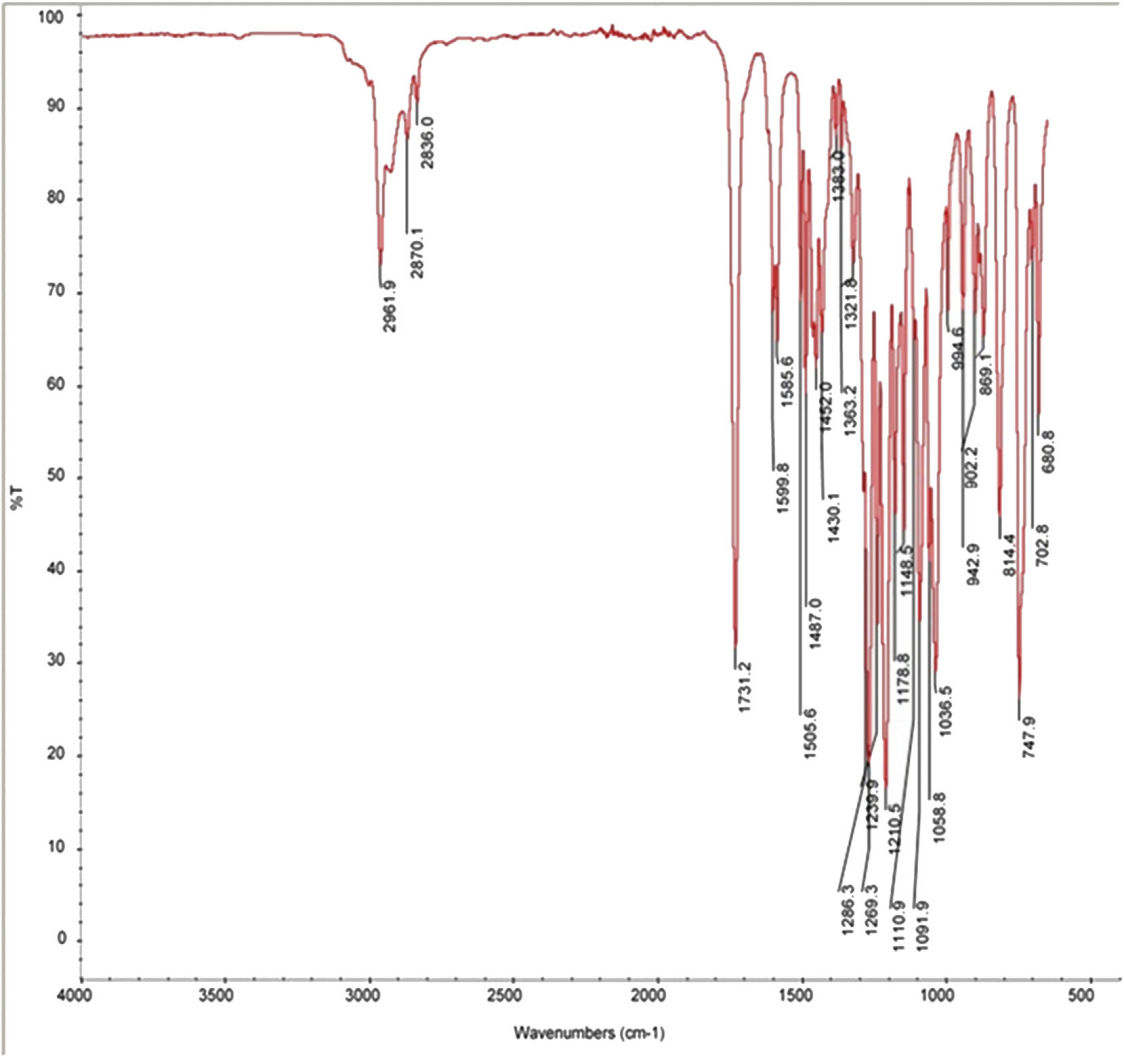
Infra-red (IR) spectra of ester-substituted derivatives of thymol.

Formation of the ester-substituted derivatives of thymol was confirmed by the absence of –OH stretching absorption of the thymol at 3310 - 3510 cm^−1^ and the presence of a strong characteristic carbonyl –C=O group at 1731.2 cm^−1^ in the IR spectra. The C - H stretching in alkyl region was characterized by absorption peaks with a shoulder at 2961.9, 2870.1 and 2836.0 cm^−1^ for the esters which is indicative of the aliphatic methylene (-CH_2_-) and methyl (-CH_3_) groups (Figure 3). Similarly, formation of the ether-substituted derivatives of thymol was confirmed by the absence of –OH stretching absorption of the thymol at 3310 - 3510 cm^−1^ and the presence of C–O group at 1255.3 cm^−1^ in the IR spectra. The C - H stretching in alkyl region was characterized by a strong absorption peaks with a shoulder at 2967.5, 2938.7 and 2879.9 cm^−1^ for the ethers which is indicative of the aliphatic methylene (-CH_2_-) and methyl (-CH_3_) groups (Figure 4).

### 3.3 Structural elucidation of ether-and-ester derivatives of thymol by using MS analysis

The synthesized thymol derivatives were characterized by using both electron ionization gas chromatography mass spectroscopy (GC-MS/EI) and chemical ionization gas chromatography mass spectroscopy (GC-MS/CI) as described below:

### 3.4 TM 2C: 2-isopropyl-5-methylphenoxy propane

The mass spectrum (EI) gave a molecular ion [M]^+^ peak of m/z 192 and a corresponding base peak at m/z 135. A characteristic tropylium ion peak at m/z 91 was also observed in the spectrum. The other prominent mass fragments for the compound are m/z 177, 150, 121, 105 and 77 as accounted for in the fragmentation pattern. The mass spectrum (CI) gave the *m/z* [M + H]^+^ and [M + C_2_H_5_]^+^ as 193 and 221 respectively.

### 3.5 TM 2D: 2-isopropyl-5-methylphenoxy methylethane

The mass spectrum (EI) gave a molecular ion [M]^+^ peak of m/z 192 and a corresponding base peak at m/z 135. A characteristic tropylium ion peak at m/z 91 was also observed in the spectrum. The other prominent mass fragments for the compound are m/z 177, 150, 121, 105 and 77 as accounted for in the fragmentation pattern. The mass spectrum (CI) gave the *m/z* [M + H]^+^ as 193.

### 3.6 TM 2E: 2-isopropyl-5-methylphenoxy 1-methylpropane

The mass spectrum (EI) gave a molecular ion [M]^+^ peak of m/z 206 and a corresponding base peak at m/z 135. A characteristic tropelium ion peak at m/z 91 was also observed in the spectrum. The other prominent mass fragments for the compound are m/z 191, 150, 121, 105, 77and 57 as accounted for in the fragmentation pattern. The mass spectrum (CI) gave the *m/z* [M + H]^+^ and [M + C_2_H_5_]^+^ as 207 and 235 respectively.

### 3.7 TM 2F: 2-isopropyl-5-methylphenoxy butane

The mass spectrum (EI) gave a molecular ion [M]^+^ peak of m/z 206 and a corresponding base peak at m/z 135. A characteristic tropelium ion peak at m/z 91 was also observed in the spectrum. The other prominent mass fragments for the compound are m/z 150, 121, 105, 77 and 57 as accounted for in the fragmentation pattern. The mass spectrum (CI) gave the *m/z* [M + H]^+^ at 207.

### 3.8 TM 2N: 2-isopropyl-5-methylphenoxy methylbenzene

The mass spectrum (EI) gave a molecular ion [M]^+^ peak of m/z 240 and a corresponding base peak at m/z 91 which is the characteristic tropylium ion peak. The other prominent mass fragments for the compound are m/z 225, 197, 149, 135, 121,105 and 77 as accounted for in the fragmentation pattern. The mass spectrum (CI) gave the *m/z* [M + H]^+^ and [M + C_2_H_5_]^+^ as 241 and 269 respectively.

### 3.9 TM 2O: 2-isopropyl-5-methylphenoxy 3-chloromethylbenzene

The mass spectrum (EI) gave a molecular ion [M]^+^ peak of m/z 274 and a corresponding base peak at m/z 125. A characteristic tropylium ion peak at m/z 91 was also observed in the spectrum. The other prominent mass fragments for the compound are m/z 259, 231, 149, 121, and 105 as accounted for in the fragmentation pattern. The mass spectrum (CI) gave the *m/z* [M + H]^+^ and [M + C_2_H_5_]^+^ as 275 and 303 respectively.

### 3.10 TM 1C: 2-isopropyl-5-methylphenyl 2-methylpropanoate

The mass spectrum (EI) gave a molecular ion [M]^+^ peak of m/z 220 and a corresponding base peak at m/z 135. A characteristic tropylium ion peak at m/z 91 was also observed in the spectrum. The other prominent mass fragments for the compound are m/z 150, 105, 71 and 43 as accounted for in the fragmentation pattern of the compound. The mass spectrum (CI) gave the *m/z* [M + H]^+^ and [M + C_2_H_5_]^+^ as 221 and 249 respectively.

### 3.11 TM 1D: 2-isopropyl-5-methylphenyl butanoate

The mass spectrum (EI) gave a molecular ion [M]^+^ peak of m/z 220 and a corresponding base peak at m/z 135. A characteristic tropylium ion peak at m/z 91 was also observed in the spectrum. The other prominent mass fragments for the compound are m/z 150, 121, 105, 71 and 43 as accounted for in the fragmentation pattern of the compound. The mass spectrum (CI) gave the *m/z* [M + H]^+^ and [M + C_2_H_5_]^+^ as 221 and 249 respectively.

### 3.12 TM 1E: 2-isopropyl-5-methylphenyl-2-methyl butanoate

The mass spectrum (EI) gave a molecular ion [M]^+^ peak of m/z 234 and a corresponding base peak at m/z 135. A characteristic tropylium ion peak at m/z 91 was also observed in the spectrum. The other prominent mass fragments for the compound are m/z 150, 115, 105, 77 and 57 as accounted for in the fragmentation pattern of the compound. The mass spectrum (CI) gave the *m/z* [M + H]^+^ and [M + C_2_H_5_]^+^ as 235 and 263 respectively.

### 3.13 TM 1F: 2-isopropyl-5-methylphenyl pentanoate

The mass spectrum (EI) gave a molecular ion [M]^+^ peak of m/z 234 and a corresponding base peak at m/z 135. A characteristic tropylium ion peak at m/z 91 was also observed in the spectrum. The other prominent mass fragments for the compound are m/z 150, 121, 105, 77 and 57 as accounted for in the fragmentation pattern of the compound. The mass spectrum (CI) gave the *m/z* [M + H]^+^ and [M + C_2_H_5_]^+^ as 235 and 263 respectively.

### 3.14 TM 1I: 2-isopropyl-5-methylphenyl 2-phenylethanoate

The mass spectrum (EI) gave a molecular ion [M]^+^ peak of m/z 268 and a corresponding base peak at m/z 135. A characteristic tropylium ion peak at m/z 91 was also observed in the spectrum. The other prominent mass fragments for the compound are m/z 150, 119, 105 and 77 as accounted for in the fragmentation pattern of the compound. The mass spectrum (CI) gave the *m/z* [M + H]^+^ and [M + C_2_H_5_]^+^ as 269 and 297 respectively.

### 3.15 TM 1Q: 2-isopropyl-5-methylphenyl 3-chlorobenzoate

The mass spectrum (EI) gave a molecular ion [M]^+^ peak of m/z 288 and a corresponding base peak at m/z 139. A characteristic tropylium ion peak at m/z 91 was also observed in the spectrum. The other prominent mass fragments for the compound are m/z 150, 111 and 77 as accounted for in the fragmentation pattern of the compound. The mass spectrum (CI) gave the *m/z* [M + H]^+^ and [M + C_2_H_5_]^+^ as 289 and 317 respectively.

### 3.16 Effect of thymol and ether-and-ester substituted derivatives on *Staphylococcus aureus*

**Figure 5:**
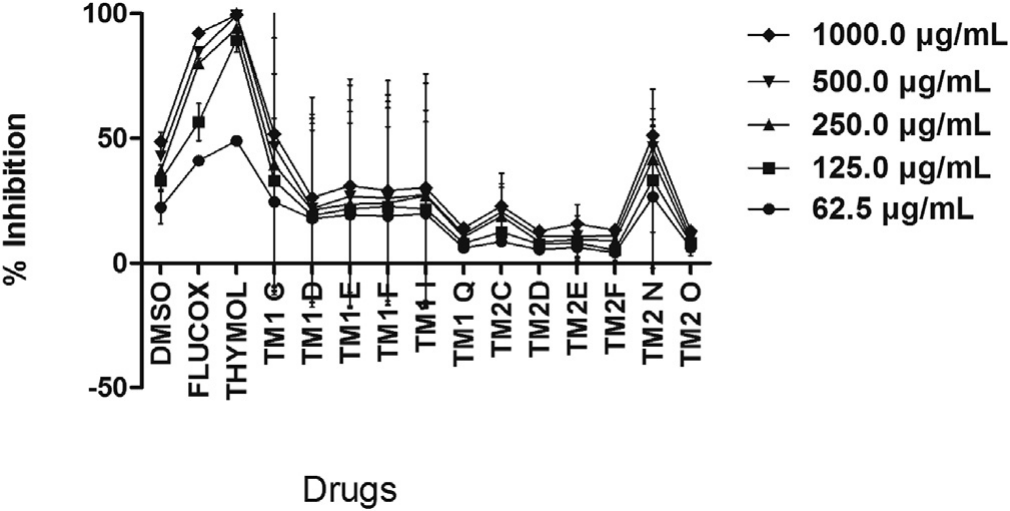
Effect of increasing concentrations (62.5–1000 μg/ml) of drugs on growth of *S. aureus.* Each point is the mean % inhibition of growth ±SD, n = 3. TM1C - 2-Isopropyl-5-methylphenyl 2-methylpropanoate, TM1D - 2-Isopropyl-5-methylphenyl butanoate, TM1E - 2-Isopropyl-5-methylphenyl-2-methyl butanoate, TM1F - 2-Isopropyl-5-methylphenyl pentanoate, TM1I - 2-Isopropyl-5-methylphenyl 2-phenylethanoate, TM1Q - 2-Isopropyl-5-methylphenyl 3-chlorobenzoate; TM2C - 2-Isopropyl-5-methylphenoxy propane, TM2D - 2-Isopropyl-5-methylphenoxy methylethane, TM2E - 2-Isopropyl-5-methylphenoxy 1-methylpropane, TM2F - 2-Isopropyl-5-methylphenoxy butane, TM2N - 2-Isopropyl-5-methylphenoxy methylbenzene, TM2O - 2-Isopropyl-5-methylphenoxy 3-chloromethylbenzene.

With MIC and MBC of >500 μg/ml, thymol was more potent against the three clinical isolates compared to both ether- and-ester substituted derivatives of thymol, which had MIC and MBC of >1000 μg/ml (Table 1). However, FLX with MIC and MBC of 62.5 μg/ml was more potent than thymol (Table 1). Thymol produced a concentration-dependent growth inhibition on *S. aureus* compared to all the twelve derivatives and the reference antibiotic FLX. Although some of the derivatives (TM2N and TM1C) produced detectable growth inhibition on *S. aureus* compared to DMSO, this was however lower than that of thymol and FLX (Figure 5). Among the six ester-substituted derivatives of thymol, TM1C produced significant growth inhibition compared to DMSO. Among the six ether-substituted derivatives of thymol, only TM2N produced a measurable growth inhibition on *S. aureus* compared to DMSO (Figure 5).

**Table 1:** Drugs and their MIC and MBC estimates on the tested clinical isolates.

DRUGS	ISOLATES					
	*S. aureus*		*E. coli*		*P. aeruginosa*	
	MIC	MBC	MIC	MBC	MIC	MBC
	(μg/ml)	(μg/ml)	(μg/ml)	(μg/ml)	(μg/ml)	(μg/ml)
THYMOL	500	500	500	500	500	500
aStreptomycin	-	-	500	500	1000	1000
bFlucloxacin	62.5	62.5	-	-	-	-
DMSO -		-	-	-	-	-
TM1C	-	-	-	-	-	-
TM1D	-	-	-	-	-	-
TM1E	-	-	-	-	-	-
TM1F	-	-	-	-	-	-
TM1I	-	-	-	-	-	-
TM1Q	-	-	-	-	-	-
TM2C	-	-	-	-	-	-
TM2D	-	-	-	-	-	-
TM2E	-	-	-	-	-	-
TM2F	-	-	-	-	-	-
TM2N -		-	-	-	-	-
TM2O	-	-	-	-	-	-

MIC – Minimum inhibitory concentration.

MBC – Minimum bactericidal concentration.

a Was not exposed to *S. aureus*.

b was not exposed to *E. coli* and *P. aeruginosa*; - had no MIC and MBC for the concentrations used in the study.

### 3.17 Effect of thymol and ether-and-ester substituted derivatives on *Pseudomonas aeruginosa*

**Figure 6:**
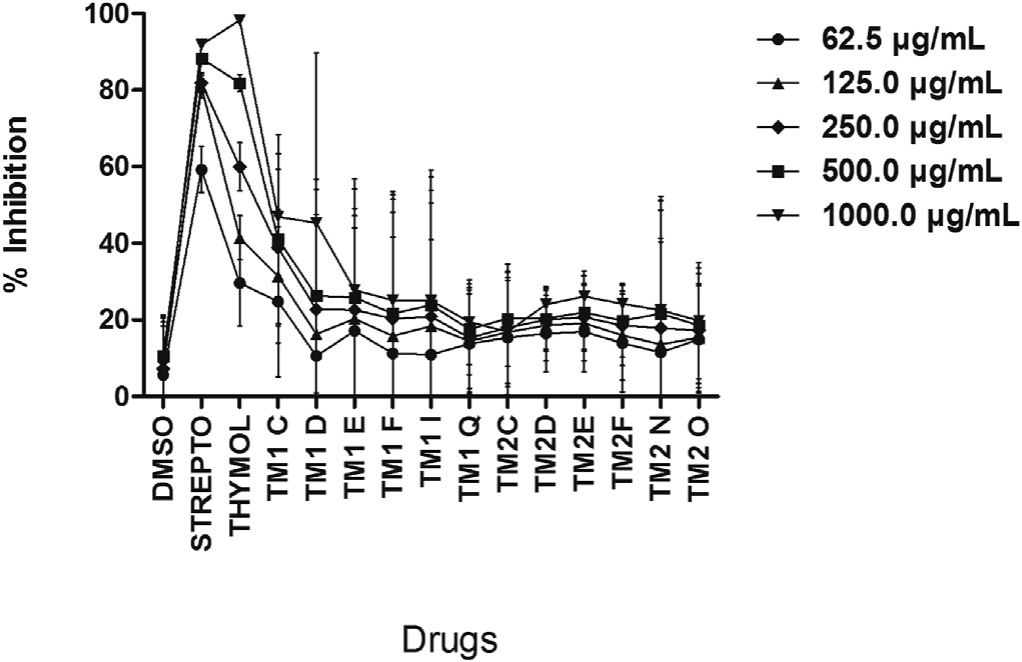
Effect of increasing concentrations (62.5–1000 μg/ml) of drugs on growth of *Pseudomonas aeruginosa*. Each point is the mean % inhibition of growth ±SD, n = 3. TM1C - 2-Isopropyl-5-methylphenyl 2-methylpropanoate, TM1D - 2-Isopropyl-5-methylphenyl butanoate, TM1E - 2-Isopropyl-5-methylphenyl-2-methyl butanoate, TM1F - 2-Isopropyl-5-methylphenyl pentanoate, TM1I - 2-Isopropyl-5-methylphenyl 2-phenylethanoate, TM1Q - 2-Isopropyl-5-methylphenyl 3-chlorobenzoate; TM2C - 2-Isopropyl-5-methylphenoxy propane, TM2D - 2-Isopropyl-5-methylphenoxy methylethane, TM2E - 2-Isopropyl-5-methylphenoxy 1-methylpropane, TM2F - 2-Isopropyl-5-methylphenoxy butane, TM2N - 2-Isopropyl-5-methylphenoxy methylbenzene, TM2O - 2-Isopropyl-5-methylphenoxy 3-chloromethylbenzene.

At equimolar concentrations, thymol and streptomycin produced concentration-dependent growth inhibition on *P. aeruginosa*; however, thymol was more potent (Figure 6 and Table 1). All the ether-substituted derivatives of thymol produced detectable growth inhibition on *P. aeruginosa* compared to DMSO, however these growth inhibitions were insignificant compared to thymol and FLX. Among the six ester-substituted derivatives of thymol, TM1C and TM1D produced concentration-dependent growth inhibition on *P. aeruginosa* compared to DMSO. None of the ether-substituted derivatives of thymol produced significant growth inhibitory effect on *P. aeruginosa* compared to DMSO (Figure 6).

### 3.18 Effect of thymol and ether-and-ester substituted derivatives on *Escherichia coli*

**Figure 7:**
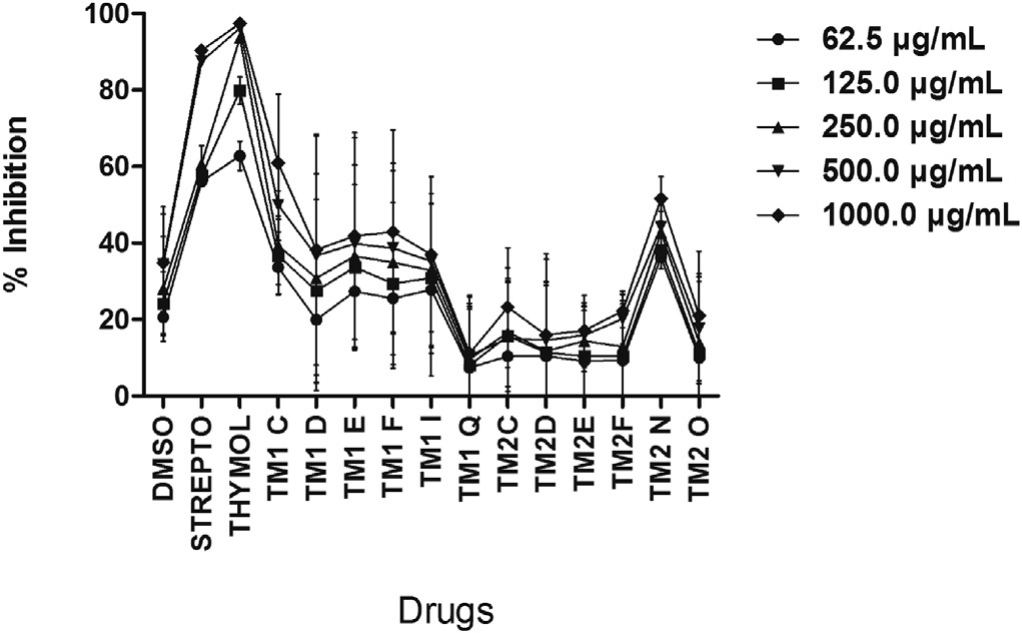
Effect of increasing concentrations (62.5–1000 μg/ml) of drugs on growth of *E. coli*. Each point is the mean % inhibition of growth ±SD, n = 3. TM1C - 2-Isopropyl-5-methylphenyl 2-methylpropanoate, TM1D - 2-Isopropyl-5-methylphenyl butanoate, TM1E - 2-Isopropyl-5-methylphenyl-2-methyl butanoate, TM1F - 2-Isopropyl-5-methylphenyl pentanoate, TM1I - 2-Isopropyl-5-methylphenyl 2-phenylethanoate, TM1Q - 2-Isopropyl-5-methylphenyl 3-chlorobenzoate; TM2C - 2-Isopropyl-5-methylphenoxy propane, TM2D - 2-Isopropyl-5-methylphenoxy methylethane, TM2E - 2-Isopropyl-5-methylphenoxy 1-methylpropane, TM2F - 2-Isopropyl-5-methylphenoxy butane, TM2N - 2-Isopropyl-5-methylphenoxy methylbenzene, TM2O - 2-Isopropyl-5-methylphenoxy 3-chloromethylbenzene.

Thymol and streptomycin produced concentration-dependent growth inhibition on *E. coli*, with thymol showing more potency than streptomycin. Except TM1Q, all (TM1I, TM1D, TMIF TM1C and, TM1E) ester-substituted derivatives of thymol produced measureable growth inhibition on *E. coli* compared to DMSO, though that of TM1C was significant (Figure 7). Among the ether-substituted derivatives of thymol, TM2N produced significant concentration-dependent growth inhibition on *E. coli* (Figure 7).

### 3.19 Side chain bulkiness of the monoterpene nucleus of thymol decreased anti-bacterial activity in corresponding ether-and-ester substituted derivatives of thymol

It was observed that among the straight-chain ester-substituted derivatives of thymol, as the side-chain increased the growth inhibitory effect of the thymol derivatives decreased (TM1D > TM1F) specifically in the case of *P. aeruginosa* and *S. aureus* (Figures 5 and 6) suggesting that the bulkiness of the side chain perhaps reduces binding of the derivative to receptor sites on the plasma membrane of the bacteria. Similarly, for the branched ester derivatives of thymol, as the side chain becomes bulkier the growth inhibitory effect decreased (TM1C > TM1E) especially in the case of *S. aureus* and *P. aeruginosa* (Figures 5 and 6). Aromatic ester-substituted derivatives of thymol produced enhanced growth inhibition than aromatic halide substituted ester derivatives of thymol particularly in the case of *P. aeruginosa* (Figure 6). Branched ester-substituted derivative of thymol (TM1C) produced broad-spectrum growth inhibition on *S. aureus*, *P. aeruginosa* and *E. coli* (Figures 5, 6, and 7).

## 4 Discussion

**Scheme 2:**
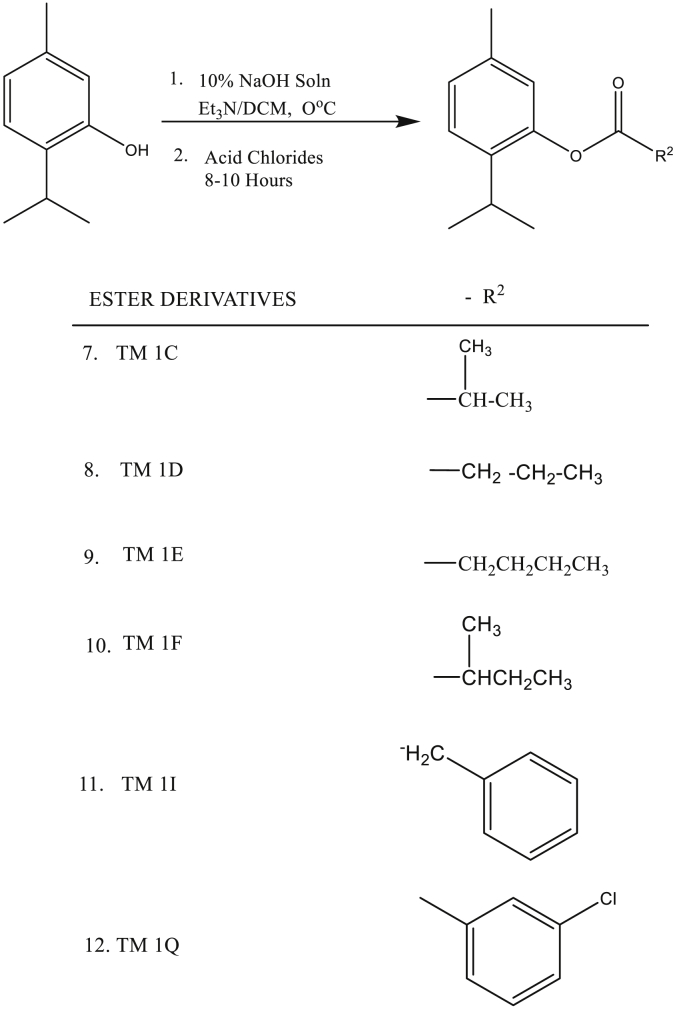
Synthesis of ester derivatives of thmol.

The utility of thymol as an anti-microbial agent in healthcare products and its potential as a template for pharmaceutical semi-synthesis is well elaborated [5, 32]. It is indicated that structural differences between monoterpene phenols (*p*-Cymene, carvacrol and thymol) do not reflect in their anti-inflammatory activity in a mice model of emphysema [15] and this observation was explained on the basis of the susceptibility of *p*-Cymene to undergo de novo biomodification to form active derivatives including thymol and carvacrol [33]. In line with this, it was speculated that oxygenation of the hydroxyl (– OH) on the monoterpene nucleus relates to enhancement of anti-microbial activity of monoterpene phenols as reviewed elsewhere [10]. Also, it is indicated that substituted phenols having log*P* greater than 2.81 [34] or bulkier alkyl substitution on the hydroxyl moiety tend to have enhanced inhibition of microbial growth [5]. On the basis of the foregoing, it is not unreasonable to expect modification of thymol specifically on the hydroxyl moiety to yield thymol derivatives that may have enhanced anti-bacterial effects against Gram-positive and Gram-negative bacteria. Previously, it was demonstrated that thymol produce bactericidal effects on *S. aureus* and *E. coli* [8]. To elucidate the anti-bacterial mechanism of thymol and related monoterpene phenols, it was shown that membrane perturbation, specifically destruction of the lipophilic fraction of plasma membrane leading to leakage and disruption of membrane structure and function was involved [6, 8, 35]. Results from the present study did not only confirm broad-spectrum anti-bactericidal effects of thymol against *S. aureus*, *P. aeruginosa* and *E. coli* as earlier reported [30, 36, 37] but also demonstrated that anti-bacterial activity of thymol, a natural monoterpene phenol is in part determined by the presence of hydroxyl –OH group on the monoterpene nucleus and that modification of the monoterpene nucleus specifically on the –OH on C_1_ with ethers (straight, branched and aromatic rings) and esters (straight, branched and aromatic rings) did not enhance anti-bacterial effects of the resultant thymol derivatives. Among the derivatives, ester-substituted derivatives of thymol produced more growth inhibition on both Gram negative and Gram positive bacteria than ether-derivatives of thymol. And that branched ester-derivatives of thymol produced enhanced growth inhibition on the test bacteria than straight-chain ester derivatives of thymol. Branched-chain ester-substituted derivatives (Scheme 2) produced enhanced growth inhibition than aromatic and straight-chain ester derivatives; this effect was bacteria type-specific. For example, TM1D produced growth inhibition specifically on *P. aeruginosa*, while TM1C produced growth inhibition on all the three isolates (*P. aeruginsoa*, *E. coli, P. aeurginosa*, and *S. aureus*), suggesting that branched ester derivatives of thymol may have broad spectrum anti-bacterial activity just like thymol.

Phenols with less bulky carbon substitutions and lower logP are shown to be less potent or display low biological activity [34]. The presents results showed that among the straight-chain ester-substituted derivatives of thymol, increase in the side-chain resulted in decreased growth inhibitory effect on all isolates as exemplified by TM1D and TM1F (Figures 5 and 6) suggesting that the bulkiness of the side chain on the –OH perhaps determines binding of the derivative to membrane receptors of pathogenic bacteria. Interestingly, this observation is not different from previously determined pattern, where increase in side-chain bulkiness and logP correlated with decrease in bioactivity of monoterpene phenols [34]. Similarly, among the branched ester-substituted derivatives of thymol, as the side chain becomes bulky the growth inhibitory effect also decreased as exemplified by TM1C and TM1E especially in the case of *S. aureus* and *P. aeruginosa* (Figures 5 and 6; Scheme 2). Substitution of a chlorine atom on the aromatic ring of the ester-substituted derivatives of thymol did not produce enhanced growth inhibition compared to aromatic ester derivatives of thymol, perhaps suggestive of lack of anti-bacterial effect of halides particularly in the case of *P. aeruginosa* (Figure 4). Among all the derivatives of thymol TM1C (Scheme 2) was outstanding in view of its broad-spectrum growth inhibition on *S. aureus*, *P. aeruginosa* and *E. coli* (Figures 5, 6, and 7), though just like the other thymol derivatives it had no MIC and MBC (Table 1) from the range of concentrations (62.5–1000 μg/ml) used in this study and this may be attributed to inoculum size effect (ESE) as previously reported [38, 39]. TM2N was the only ether derivative which showed anti-bacterial effect on *S. aureus* and *E. coli* but poorly on *P. aeruginosa*, indicating that ether-substituted derivatives of thymol may be effective against Gram-positive bacteria and Gram-negative lactose fermenters. It is quite obvious that the potency of the ether-and-ester substituted derivatives of thymol on the clinical isolates is related to the presence of an aromatic ring attached to the alkyl side chain of the ether functional groups as was evident in TM2N which exhibited the highest activity among the ether-substituted derivatives of thymol. The contribution of an aromatic nucleus in the activity of the ester-substituted derivatives (TM 1I and TM 1Q) was minimal. Also, the number of carbon atoms up to a certain limit in the side chain of both the ether-and-ester functional groups contributed to their activity. For instance, activity was relatively higher with three carbons at the side chain but decreases with carbon number above three in the carbon chain. Similarly, the nature of the aliphatic side chain contributed to anti-bacterial activity, as branching of the alkyl chain resulted in a decrease in anti-bacterial activity in the ether-substituted derivatives; there was however an increase in anti-bacterial activity of the ester-substituted derivatives as a result of the degree of branching in the alkyl chain. This was evident in TM 1C among the ester-substituted derivatives with the highest anti-bacterial effect against *S. aureus.* Generally, the potency of the ester-substituted derivatives on the *S. aureus* was fairly higher than the corresponding ether-substituted derivatives except TM 2N which exhibited the highest activity among all the twelve thymol derivatives. The contribution of the weakly deactivating Cl group substituted on the aromatic nucleus of the ester (TM 1Q) and the ether (TM 2O) derivatives had no measurable contribution to anti-bacterial activity on *S. aureus*. Synthesis, characterization and *in vitro* anti-bacterial activity of TM IC, TM ID and TM 1I have been reported [28], however, the present results only supports TM1C but not the others. Also, synthesis and anti-fungal effects of TM 2C, TM 2D, TM 2F and TM 2N have been reported [40] but the present results indicate that the anti-microbial effects of the ether-substituted derivatives of thymol show pathogen-specific anti-microbial activity. However, synthesis, characterization and *in vitro* anti-bacterial activity of TM 2O, TM 1F and TM 1Q are being reported for the first time. The study was limited by the unavailability of proton nuclear magnetic resonance (^1^H-NMR) and carbon-13 nuclear magnetic resonance (^13^C-NMR) spectral data on the unknown compounds (TM 2O, TM 1F and TM 1Q), which our follow up study will address. Notwithstanding, the present study provides an important basis for future efforts to chemically explore the possibility of modifying the monoterpene nucleus to optimize specific bioactivity of thymol in essential oils.

## 5 Conclusion

Thymol has demonstrated broad-spectrum anti-bacterial effects attributable to the hydroxyl moiety on C1 of the monoterpene nucleus. Structural modification of the hydroxyl moiety on C1 of the monoterpene nucleus of thymol with either ether or ester substitutions yielded no significant anti-bacterial effects. As the current result provides basis for further exploitation of chemical modification of the –OH on the C1 of the monoterpene nucleus of thymol, possibly moving the –OH to other carbons (C3 or C4), it also demonstrates that the food and pharmaceutical industries can rely on thymol from naturally occurring essential oils as a natural/organic preservative.

## Declarations

### Author contribution statement

Alex Boye: Conceived and designed the experiments; Wrote the paper.

Justice Kwaku Addo: Conceived and designed the experiments; Performed the experiments.

Desmond Omane Acheampong: Analyzed and interpreted the data; Contributed reagents, materials, analysis tools or data; Wrote the paper.

Ama Kyeraa Thomford: Performed the experiments; Contributed reagents, materials, analysis tools or data.

Dominic Nkwantabisa Kuma, Emmanuel Asante and Regina Elorm Amoaning: Performed the experiments.

### Funding statement

This research did not receive any specific grant from funding agencies in the public, commercial, or not-for-profit sectors.

### Competing interest statement

The authors declare no conflict of interest.

### Additional information

No additional information is available for this paper.
